# On the Use of Glycerine in Surgery and Medicine

**Published:** 1863-06

**Authors:** E. J. Tilt


On the Use of Glycerine in Surgery and Medicine. *By E. J. Tilt, M.D., M.R.C.P., etc.*—Glycerine is not sufficiently valued in this country as a therapeutical agent; whereas the high estimation in which it is held on the Continent may be inferred from the fact, that from 1851 to 1861 the annual consumption of glycerine in the Paris hospitals rose from 300 lbs. to 3000 lbs. I propose to point out the principal advantages of glycerine from personal experience, and from that of an eminent Paris surgeon, M. Demarquay, who has been chiefly instrumental in introducing this agent, and has just published the results of his experience in an interesting little work. In the process of making lead-plaster, glycerine is produced, but, as it contains lead, it has an irritating action on abraded surfaces. The only glycerine, therefore, fit for medical and surgical uses is Price's, which is made by subjecting palm oil to steam raised to a temperature of 300° centigrade, and its specific gravity should be 1·26.

Glycerine is too well known to require description. Although derived from fatty substances, it will not combine with them, but mixes with water in any proportion, and has the power of dissolving all our active therapeutical agents about as readily as weak alcohol.

### SURGICAL USES OF GLYCERINE.

*Pure Glycerine.*—In household surgery, glycerine is known as the best remedy for chapped hands and slight irritation of the face and lips. I have found it invaluable when freely used in nasal, pudental, and anal irritation. It is applied in a large number of skin diseases in France; and Maisonneuve, Denonvilliers and Demarquay use it to dress ulcers and wounds, instead of cerate. It appears to have antiseptic properties, inasmuch as it speedily gives a healthy appearance to foul, unhealthy, and even pultaceous-looking wounds. This is admitted by Baron Larrey, whose report is in other respects unfavorable to its use in surgery. Indeed, this antiseptic property might be inferred from its preserving from decomposition meat and microscopic objects that are kept in it, or have been steeped in it.

*Liniments.*—Glycerine does not become rancid, like oil. It is cleaner, can be easily washed off, and does not stain the body linen like oil. Though glycerine does not dissolve fat, it is said to dissolve fat, it is said to dissolve the sebaceous product of the skin, and thereby to facilitate the absorption of the various ingredients which it may hold in solution. For these reasons, glycerine is far preferable to oil as a basis for liniments.

*Lotions.*—Since Mr. Startin has praised glycerine as a useful ingredient of lotions for the skin, this has been fully admitted. Its stability, cleanliness, innocuousness, and antiseptic properties make it a valuable ingredient for all the variety of lotions which are applied to the inflamed or the unhealthy mucous membranes of the mouth, eyes, nose, ears, rectum and vagina.

*Ointments.*—When starch is boiled in glycerine the membranes burst and uniformly thicken the liquid. If eighty grains of starch are boiled in one fluid ounce of glycerine, a moderately stiff and tenacious plasma or ointment is the result. It is stable, inodorous, clean, and is capable of holding in solution or suspension all the agents usually incorporated with lard. Glycerine ointment does not become rancid like other fatty substances, does not soil the body linen, and can be instantaneously removed by means of a damp towel.

Mr. Bullock, of Hanover street, has made experiments of combining glycerine with several kinds of starch, and I lately exhibited the samples at a meeting of the Obstetrical Society.

Every kind of starch makes a very serviceable product, but maize and the ordinary starch seem to give the stiffest and most satisfactory result.

It has been objected to glycerine ointment that it is too absorbent of moisture to be useful; but this is not true. It absorbs moisture only to a limited extent—suggesting to the pharmaceutist the advisability of not making a large quantity at a time, and of keeping it closely covered up.

Glycerine ointment has been used in France under the name of *glycerat d'amidon.* It has been extensively prescribed by my friend Mr. Henry Lee, and by Dr. Symonds and Dr. W. Budd, of Clifton. Mr. Schacht, of the same town, wrote a paper on the subject, which will be found in the *Pharmaceutical Journal* for 1858. For the pelvic and spinal pains attendant on uterine inflammation, I frequently prescribe the following ointments:—Sulphate of atropia, two grains; glycerine, half a drachm; oil of neroli, four drops glycerine ointment, one ounce. A portion of this ointment about the size of a small walnut, is to be well rubbed in, night and morning. Acetate of morphia, ten grains; otto of roses, one drop; glycerine, half a drachm; glycerine ointment, one ounce.

*Plasters.*—It occurred to me that by boiling a larger quantity of starch in the same quantity of glycerine the ointment might become stiff enough for all the purposes of plasters. Mr. Bullock, therefore, boiled 100 to 150 grains of starch in an ounce of glycerine, and obtained a very firm and tenacious compound, to which I have directed attention, in my "Handbook of Uterine Therapeutics," as well calculated to make ready-made plasters, not open to the objections raised against those in common use, which either do not stick at all, or stick so firmly that their removal is difficult. Some of them also smell so disagreeable as to interfere with a patient's sleep, while others cause a skin irritation which was not desired. With the glycerine plasters the patient may continue using the sponge bath or any other bath that may be advisable, as there is no difficulty in removing and replacing the application. This hard glycerine ointment is capable of holding, partly in solution partly in suspension, all the ingredients of the plasters now in use. It can be made softer by being rubbed up with a little glycerine, and I tell the patient to spread it thickly with a paper-knife on a gutta-percha cloth, or on the fluffy side of leather, or on impermeable wash leather. Before re-applying the plaster it is well to spread a little more ointment, and they can be speedily cleaned with a sponge and tepid water. Thus, instead of prescribing a belladonna plaster, I order—Sulphate of atropia, four grains; otto of roses, one drop; hard glycerine ointment, one ounce. The salt is to be rubbed down with a few drops of glycerine, and incorporated with the ointment. I give veratria in the proportion and double the quantity of morphia, following compound sedative plaster can be made in the same manner:—Sulphate of atropia, three grains; veratria, three grains; sulphate of morphia, eight grains; otto of roses, one drop; hard glycerine ointment, one ounce.

MEDICINAL USE OF GLYCERINE.

It is said to be useful in dysentery, and its antiseptic properties justify its trial in cases of ulceration and inflammation of the stomach and intestines. Experience has not confirmed the assertions of those who affirm that it acts on the system like cod-liver oil.—*London Lancet.*

